# Association between dietary fatty acids and all‐cause mortality in patients with cirrhosis: A cohort study

**DOI:** 10.1113/EP093526

**Published:** 2026-09-28

**Authors:** Ali Jafari, Ghazaleh Mahdavi‐Mazdeh, Behnaz Narimani, Helia Mardani, Azita Hekmatdoost, Fereshteh Pashayee‐Khamene, Sara Karimi, Saleheh Ahmadzadeh, Mehdi Saberifiroozi, Behzad Hatami, Zahra Yari

**Affiliations:** ^1^ Student Research Committee, Faculty of Nutrition Sciences and Food Technology, National Nutrition and Food Technology Research Institute Shahid Beheshti University of Medical Sciences Tehran Iran; ^2^ Clinical Nutrition and Dietetics Department Faculty of Nutrition Sciences and Food Technology National Nutrition and Food Technology Research Institute Shahid Beheshti University of Medical Sciences Tehran Iran; ^3^ Students' Scientific Research Center (SSRC) Tehran University of Medical Sciences Tehran Iran; ^4^ Student Research Committee Ahvaz Jundishapur University of Medical Sciences Ahvaz Iran; ^5^ Liver and Pancreatobiliary Research Center, Digestive Disease Research Institute Shariati Hospital, Tehran University of Medical Sciences Tehran Iran; ^6^ Gastroenterology and Liver Diseases Research Center, Research Institute for Gastroenterology and Liver Diseases Shahid Beheshti University of Medical Sciences Tehran Iran; ^7^ Department of Nutrition Research, National Nutrition and Food Technology Research Institute and Faculty of Nutrition Sciences and Food Technology Shahid Beheshti University of Medical Sciences Tehran Iran

**Keywords:** dietary fatty acids, liver cirrhosis, mortality, omega‐3, omega‐6, prospective cohort, saturated fat

## Abstract

Liver cirrhosis imposes a substantial global burden, with complications driving high mortality. Diet is pivotal in managing liver disease progression and cirrhosis‐related death. This study investigated associations between total fat intake, specific dietary fatty acid subclasses and all‐cause mortality in cirrhotic patients. In this prospective cohort study, 121 ambulatory patients with recently diagnosed cirrhosis (≤6 months) were enrolled. Baseline dietary intake was evaluated via a validated 168‐item semi‐quantitative food frequency questionnaire. Participants were categorized into tertiles by total fat and fatty acid subclasses: saturated (SFA), monounsaturated (MUFA), polyunsaturated (PUFA), omega‐3 and omega‐6 fatty acids. Cox proportional hazards models computed hazard ratios (HRs) and 95% confidence intervals (CIs), adjusted sequentially for age, sex, energy intake, BMI, smoking, alcohol use, cirrhosis aetiology, MELD score and Child–Pugh class. During the 60‐month follow‐up, 50 deaths (41.3%) occurred. In fully adjusted models, highest versus lowest tertiles of total fat intake (HR = 6.69; 95% CI: 1.37–13.34; *P*‐trend = 0.017), SFA intake (HR = 8.2; 95% CI: 1.33–20.53; *P*‐trend = 0.014) and PUFA‐6 intake (HR = 5.24; 95% CI: 1.1–26.44; *P*‐trend = 0.014) were independently associated with significantly elevated mortality risk. No significant associations were observed for MUFA, total PUFA or PUFA‐3 intake. Kaplan–Meier analyses confirmed significantly reduced 5‐year survival with higher total fat (log‐rank *P* = 0.003) and SFA intake (log‐rank *P *< 0.001). In this exploratory prospective cohort study, higher intakes of total fat, saturated fatty acids and omega‐6 polyunsaturated fatty acids were associated with increased all‐cause mortality among patients with cirrhosis. These preliminary findings are hypothesis‐generating and require confirmation in larger, adequately powered prospective studies.

## Abstract (highlights)

- **What is the central question of this study?**
  Is habitual dietary intake of specific fatty acid subclasses (saturated, monounsaturated, polyunsaturated fatty acids) associated with all‐cause mortality risk in patients with established liver cirrhosis?
- **What is the main finding and its importance?**
  Higher intakes of saturated fatty acids and omega‐6 polyunsaturated fatty acids were independently associated with increased all‐cause mortality, while monounsaturated, total polyunsaturated and omega‐3 fatty acids showed no association. These hypothesis‐generating findings highlight dietary fatty acid composition as a potential modifiable factor that warrants confirmation in larger prospective studies.

## 1 INTRODUCTION

Liver cirrhosis represents a substantial global health burden, affecting an estimated 160 million individuals worldwide and accounting for more than 1.4 million deaths annually (Ginès et al., 2021; Piano et al., 2017; Zamani et al., 2025). This chronic, progressive disease arises from diverse aetiologies including viral hepatitis, alcohol‐related liver disease, metabolic dysfunction‐associated steatotic liver disease, autoimmune disorders and cholestatic conditions (Ginès et al., 2021; Piano et al., 2017). Epidemiological projections indicate that the incidence of decompensated cirrhosis and associated mortality will continue to rise over the ensuing decade (Huang et al., 2023). The clinical spectrum encompasses wide‐ranging manifestations, from asymptomatic presentation to life‐threatening complications including ascites, variceal haemorrhage, hepatic encephalopathy and progressive sarcopenia (Garcia‐Tsao et al., 2010). Notably, cardiovascular complications affect more than half of cirrhotic patients and constitute a leading cause of death in this population (Kaur & Premkumar, 2022).

Dysregulated fatty acid metabolism occupies a central position in the pathophysiology of chronic liver disease and its progression to cirrhosis (Ginanni Corradini et al., 2014; Habibullah et al., 2024; Musso et al., 2018). Aberrant lipid accumulation in hepatocytes initiates a cascade of pathological processes, including mitochondrial dysfunction, endoplasmic reticulum stress, oxidative damage and activation of inflammatory pathways, ultimately culminating in hepatocyte injury and fibrogenesis (Méndez‐Sánchez et al., 2024; Rao et al., 2023). Clinical evidence demonstrates that plasma fatty acid profiles undergo marked alterations in cirrhotic patients, with certain fatty acid species correlating with disease severity and clinical outcomes (Ginanni Corradini et al., 2014; Liu et al., 2024). Current nutritional guidelines recommend daily total fat intake comprising 20–35% of total energy, with specific recommendations for constituent fatty acid subclasses: monounsaturated fatty acids (MUFAs) 10–25%, polyunsaturated fatty acids (PUFAs) 6–11% and saturated fatty acids (SFA) less than 10% (Musso et al., 2018). However, emerging evidence suggests that fatty acid quality may exert more profound effects on metabolic and hepatic health than total fat quantity alone (Gillingham et al., 2011; Kromhout et al., 2010; Schmitz & Ecker, 2008). Experimental investigations have yielded inconsistent findings, with animal studies demonstrating that MUFAs may exert favourable effects on hepatic histology compared with PUFAs (Fernandez et al., 1997), while others report conflicting outcomes regarding unsaturated fatty acid supplementation (Fernández et al., 2005; Moreira et al., 1995). Recent epidemiological studies examining dietary fat composition and liver disease outcomes have produced inconsistent results, with some associations varying by underlying aetiology (Fridén et al., 2024; Kirpich et al., 2016).

Despite the established metabolic perturbations in cirrhotic patients and the biological plausibility linking dietary fatty acid intake to disease outcomes, the relationship between specific dietary fatty acid classes and mortality risk among patients with cirrhosis remains largely unexplored. To our knowledge, no prospective cohort study has systematically examined the associations between habitual intake of different fatty acid subclasses and all‐cause mortality in patients with established liver cirrhosis. Given the limited prior evidence on this topic, this study was designed as an exploratory (hypothesis‐generating) prospective cohort study to investigate potential associations between dietary fatty acid intake patterns and the risk of all‐cause mortality among adults with liver cirrhosis. Understanding these relationships may inform evidence‐based nutritional recommendations and targeted dietary interventions to improve survival outcomes in this vulnerable patient population.

## 2 METHODS

### 2.1 Study design and population

This prospective cohort investigation enrolled 166 ambulatory patients with recently diagnosed liver cirrhosis (within 6 months of diagnosis) from two tertiary‐care teaching hospitals in Tehran, Iran, with recruitment occurring between 2016 and 2018. The methodological framework and cohort characteristics have been comprehensively detailed in our prior publications from this research initiative (Fotros et al., 2025; Tabar et al., 2025). Participants underwent systematic follow‐up extending through 30 April 2023, yielding a maximum observation period of 60 months.

We implemented a structured annual telephone‐based surveillance protocol to document vital status and intercurrent medical events. Individuals meeting any of the following criteria were deemed ineligible: pregnancy or lactation; concurrent diagnoses of diabetes mellitus, malignancy, renal insufficiency, cardiac pathology, infectious processes, pancreatic dysfunction or acquired immunodeficiency syndrome. Additional exclusions were applied during analytical phases for participants demonstrating: (1) reported energy intake beyond physiologically plausible thresholds (<500 or >5000 kcal/day, representing values exceeding ±3 standard deviations from the cohort mean); (2) incident cancer detection during the initial 12 months; (3) insufficient completion of dietary or lifestyle questionnaires; or (4) extreme body mass index measurements (<15 or >50 kg/m^2^). Following application of these criteria, 121 patients (83 males, 38 females) constituted the final analytical cohort.

The investigation received approval from the institutional review board of the National Nutrition and Food Technology Research Institute (NNFTRI) under protocol IR.SBMU.NNFTRI.1396.186. After the study protocol was detailed, all enrolled individuals provided written informed consent prior to participation.

### 2.2 Assessment of dietary fatty acid intake

Habitual dietary consumption patterns were captured at enrolment through structured face‐to‐face interviews implementing a previously validated 168‐item semi‐quantitative food frequency questionnaire (FFQ) developed and validated for the Iranian population (Mirmiran et al., 2010). The timing of dietary assessment preceded substantial disease‐related modifications to eating patterns. Trained registered dietitians conducted all interviews, providing standardized visual aids and portion size references to enhance recall accuracy.

Participants reported consumption frequency for each food item across the preceding 12‐month period, with responses recorded on daily, weekly or monthly scales. These frequencies were subsequently converted to daily gram equivalents utilizing Iranian household measures and standard portion sizes (Azar & Sarkisian, 1980). Nutrient composition analysis was performed using Nutritionist IV software (version 4.1; First Databank, San Bruno, CA, USA), with nutrient values derived from the United States Department of Agriculture (USDA) Food Composition Database.

For the current analysis, we quantified total dietary fat intake alongside constituent fatty acid subclasses: SFAs, MUFAs and PUFAs, with PUFAs further categorized into omega‐3 (PUFA‐3) and omega‐6 (PUFA‐6) subclasses. All fatty acid exposures were expressed as grams per day and subsequently adjusted for total energy intake using the residual method.

### 2.3 Clinical and anthropometric measurements

Comprehensive baseline data collection encompassed demographic variables (age, sex), lifestyle factors (tobacco use defined as >1 cigarette daily; alcohol consumption defined as >20 g ethanol daily), cirrhosis aetiology (viral, autoimmune, other) and clinical disease severity indicators. Anthropometric evaluations followed standardized protocols with participants measured in minimal clothing without footwear. Body weight was recorded using calibrated digital scales to the nearest 100 g, while standing height was measured with a portable stadiometer to the nearest 0.1 cm. Body mass index was computed as weight (kg) divided by height squared (m^2^). Nutritional status was systematically evaluated using the Subjective Global Assessment (SGA) instrument, yielding three categorical classifications: A (well‐nourished), B (mild‐to‐moderate malnutrition) and C (severe malnutrition) (Detsky et al., 1987). Cirrhosis severity and prognostic stratification were accomplished through calculation of the Child–Pugh classification (stages A, B, C) incorporating serum bilirubin, albumin, prothrombin time, and clinical assessment of ascites and hepatic encephalopathy (Malinchoc et al., 2000). Additionally, the Model for End‐Stage Liver Disease (MELD) score was calculated using the validated formula incorporating serum creatinine, bilirubin and international normalized ratio (INR) (Malinchoc et al., 2000).

### 2.4 Statistical analysis

Participants were stratified into tertiles based on total dietary fat consumption for baseline comparisons. Distribution of categorical variables across tertiles was evaluated using Pearson's chi‐square (χ^2^) test, while continuous variables were analysed via one‐way analysis of variance (ANOVA) with appropriate *post hoc* testing. The primary outcome was all‐cause mortality. Person‐time was calculated from enrolment date until death occurrence, loss to follow‐up, or administrative censoring on 30 April 2023. Cox proportional hazards regression models were constructed to estimate hazard ratios (HRs) and corresponding 95% confidence intervals (CIs) for the association between fatty acid intake tertiles and mortality risk. The proportional hazards assumption was verified using Schoenfeld residuals.

Three sequential multivariable adjustment models were specified to control for potential confounding: Model 1 adjusted for age (continuous) and sex (male/female); Model 2 additionally incorporated energy intake (continuous), body mass index (continuous), smoking status (yes/no) and alcohol consumption (yes/no); Model 3 further adjusted for cirrhosis aetiology (viral/autoimmune/other), MELD score (continuous) and Child–Pugh classification (A/B/C). Linear trend tests across tertiles were performed by modelling the median value of each tertile as a continuous variable.

Kaplan–Meier survival curves were generated to visualize cumulative survival probabilities across fatty acid intake tertiles, with between‐group differences assessed using the log‐rank test. The proportional hazards assumption was evaluated using Schoenfeld residuals, and no violations were detected for any of the covariates or for the global test. All statistical procedures were executed using SPSS Statistics version 19.0 (IBM Corp., Armonk, NY, USA). Two‐tailed *P*‐values <0.05 were considered statistically significant.

## 3 RESULTS

### 3.1 Baseline characteristics

Table 1 presents the baseline characteristics of the 121 study participants stratified by tertiles of total dietary fat intake. The cohort comprised predominantly male patients (68.6%; *n* = 83), with a mean age of 55 ± 12 years. Across the tertiles, mean total fat intake was 38 ± 9, 59 ± 5 and 85 ± 14 g/day for T1, T2 and T3, respectively (*P* < 0.001). Although sex distribution differed across fat‑intake tertiles, all models were adjusted for sex, and energy adjustment using the residual method further minimized sex‑related variation in dietary intake. These factors make substantial residual confounding by sex unlikely.

**TABLE 1:** Characteristics of participants according to the fat intake tertile.

	Tertile of total fat intake			
	T1	T2	T3	*P*
Men (%)	41	74	80	0.001
Age (years)	55 ± 11	54 ± 14	56 ± 10	0.789
Aetiology of cirrhosis				0.189
Virus	44	52	65	
Autoimmune	50	32	23	
Other	6	16	12	
MELD score	13.1 ± 6	12 ± 5	11.3 ± 4	0.400
Child–Pugh category (A/B/C) (%)				0.229
A	61.5	76.7	81.5	
B, C	38.5	23.3	18.5	
Alcohol drinker (%)	18.2	17.6	22.6	0.861
Smoker (%)	30.3	34.3	43.8	0.511
Weight (k)g	71.4 ± 15.8	73.8 ± 20.1	74.9 ± 14.4	0.473
Height (cm)	160.5 ± 7.6	166.2 ± 7.9	168.5 ± 8.6	0.001
Body mass index (kg/m^2^)	26.8 ± 5.7	26.7 ± 6	27.8 ± 4.7	0.621
Subjective global assessment				0.155
A	17.6	40	37.1	
B	70.6	42.9	45.7	
C	11.8	17.1	17.1	
Calorie intake (kcal/day)	1610 ± 549	2195 ± 483	2513 ± ‌ 513	<0.001
Total fat intake (g/day)	38 ± 9	59.3 ± 5.5	85 ± 14	<0.001
SFA (g/day)	11.9 ± 3.9	18.5 ± 6.9	24.9 ± 7.6	<0.001
MUFA (g/day)	15.3 ± 9.7	23.3 ± 8.4	29.7 ± 8.6	<0.001
PUFA (g/day)	9.6 ± 4	14.1 ± 4.3	20.6 ± 4.8	<0.001
PUFA‐6 (g/day)	2.8 ± 2.4	3.9 ± 2.6	4.4 ± 2.7	0.037
PUFA‐3 (g/day)	0.5 ± 0.3	0.8 ± 0.4	1.2 ± 0.6	<0.001
Cholesterol (mg/day)	120 ± 70	187 ± 71	212 ± 77	<0.001

*Note*: Values are means ± SD for continuous variables and percentages for categorical variables. ANOVA for quantitative variables and χ^2^ test for qualitative variables.

Significant sex distribution differences emerged across fat intake tertiles, with males comprising 41%, 74% and 80% of participants in T1, T2 and T3, respectively (*P* = 0.001). Mean height also differed significantly across tertiles (160.5 ± 7.6, 166.2 ± 7.9 and 168.5 ± 8.6 cm for T1, T2 and T3, respectively; *P* = 0.001). No statistically significant differences were observed across tertiles for age (*P* = 0.789), body weight (*P* = 0.473), BMI (*P* = 0.621), cirrhosis aetiology (*P* = 0.189), MELD score (*P* = 0.400), Child–Pugh classification (*P* = 0.229), SGA category (*P* = 0.155), alcohol consumption (*P* = 0.861) or smoking status (*P* = 0.511).

Daily energy intake increased progressively with higher fat consumption tertiles (1610 ± 549, 2195 ± 483 and 2513 ± 513 kcal/day for T1, T2 and T3, respectively; *P *< 0.001). Similarly, all fatty acid subclasses demonstrated significant increases across tertiles: SFA (11.9 ± 3.9, 18.5 ± 6.9 and 24.9 ± 7.6 g/day; *P *< 0.001), MUFA (15.3 ± 9.7, 23.3 ± 8.4 and 29.7 ± 8.6 g/day; *P *< 0.001), total PUFA (9.6 ± 4.0, 14.1 ± 4.3 and 20.6 ± 4.8 g/day; *P *< 0.001), PUFA‐3 (0.5 ± 0.3, 0.8 ± 0.4 and 1.2 ± 0.6 g/day; *P *< 0.001) and PUFA‐6 (2.8 ± 2.4, 3.9 ± 2.6 and 4.4 ± 2.7 g/day; *P* = 0.037). Dietary cholesterol intake also increased significantly across tertiles (120 ± 70, 187 ± 71 and 212 ± 77 mg/day; *P *< 0.001).

Appendix Table A1 presents baseline protein intake across fat‐intake tertiles and the results of a sensitivity model additionally adjusted for total protein intake. Protein intake did not differ significantly across tertiles and did not alter the associations between dietary fat subclasses and mortality.

### 3.2 Total fat intake and all‐cause mortality

During the 60‐month follow‐up period, 50 deaths (41.3%) occurred among the 121 participants. Mortality events increased with higher total fat intake, with 11 deaths (27.5%) in T1, 16 deaths (38.1%) in T2 and 23 deaths (57.5%) in T3 (*P*‐trend = 0.031) (Table 2).

**TABLE 2:** Hazard ratios for total mortality, according to the fat intake tertile.

	Tertiles of fat intake			
	T1	T2	T3	*P*‐trend
**Total fat**				
Intake (g/day)	<50.7	50.7–68.9	≥68.9	
No. of deaths	11	16	23	0.031
HR (95% CI)				
Model 1	ref	1.2 (0.48–2.99)	2.31 (1.09–5.65)	0.041
Model 2	ref	1.34 (0.44–4.02)	4.26 (1.28–14.16)	0.007
Model 3	ref	0.6 (0.13–2.67)	6.69 (1.37–13.34)	0.017
**SFA**				
Intake (g/day)	<15.2	15.2–22.2	≥22.2	
No. of deaths	10	14	26	0.001
HR (95% CI)				
Model 1	ref	1.26 (0.54–2.88)	3.56 (1.63–7.75)	0.001
Model 2	ref	2.01 (0.77–5.25)	10.4 (2.91–22.43)	0.005
Model 3	ref	1.91 (0.48–7.66)	8.2 (1.33–20.53)	0.014
**MUFA**				
Intake (g/day)	<17.8	17.8–30.5	≥30.5	
No. of deaths	20	15	15	0.559
HR (95% CI)				
Model 1	ref	0.76 (0.36–1.6)	0.82 (0.39–1.69)	0.631
Model 2	ref	0.83 (0.35–1.95)	0.64 (0.21–1.89)	0.418
Model 3	ref	0.47 (0.13–1.75)	0.57 (0.13–2.45)	0.470
**PUFA**				
Intake (g/day)	<12	12–19.3	≥19.3	
No. of deaths	15	14	21	0.212
HR (95% CI)				
Model 1	ref	0.85 (0.41–1.76)	1.02 (0.50–2.06)	0.932
Model 2	ref	0.93 (0.42–2.11)	1.74 (0.59–5.03)	0.414
Model 3	ref	0.923 (0.31–2.75)	1.48 (0.37–5.88)	0.625
**PUFA‐6**				
Intake (g/day)	<2.4	2.4–4.8	≥4.8	
No. of deaths	6	15	29	0.001
HR (95% CI)				
Model 1	ref	1.43 (0.55–3.7)	3.46 (1.4–8.4)	0.001
Model 2	ref	1.24 (0.43–3.6)	4.03 (1.45–11.2)	0.005
Model 3	ref	1.29 (0.27–6.17)	5.24 (1.1–26.44)	0.014
**PUFA‐3**				
Intake (g/day)	<0.59	0.59–1.15	≥1.15	
No. of deaths	20	18	12	0.152
HR (95% CI)				
Model 1	ref	0.74 (0.35–1.57)	0.8 (0.42–1.52)	0.404
Model 2	ref	0.5 (0.18–1.41)	0.54 (0.22–1.34)	0.227
Model 3	ref	0.54 (0.14–2.03)	0.78 (0.24–2.49)	0.346

*Note*: Cox proportional hazards regression models for estimating HRs and 95% CIs. Model 1: adjusted for age and sex. Model 2: additionally adjusted for energy intake, BMI, smoking and alcohol. Model 3: additionally adjusted for aetiology, MELD and child.

In Model 1 (adjusted for age and sex), participants in T3 demonstrated significantly elevated mortality risk compared with T1 (HR = 2.31; 95% CI: 1.09–5.65), while T2 showed a non‐significant trend (HR = 1.2; 95% CI: 0.48–2.99; *P*‐trend = 0.041). After additional adjustment for energy intake, BMI, smoking and alcohol consumption in Model 2, the association strengthened substantially, with T3 showing markedly increased risk (HR = 4.26; 95% CI: 1.28–14.16; *P*‐trend = 0.007). In the fully adjusted Model 3 (additionally controlling for cirrhosis aetiology, MELD score and Child–Pugh classification), the highest tertile maintained a significant positive association with mortality (HR = 6.69; 95% CI: 1.37–13.34; *P*‐trend = 0.017), while T2 demonstrated a non‐significant inverse association (HR = 0.6; 95% CI: 0.13–2.67). To evaluate whether protein intake confounded the association between dietary fat and mortality, we conducted a sensitivity analysis by adding total protein intake to the fully adjusted Cox model (Table A2). The associations between total fat intake and mortality remained significant and materially unchanged after additional adjustment for protein.

### 3.3 Fatty acid subclasses and mortality risk

#### 3.3.1 SFAs

Higher SFA intake demonstrated a strong dose–response relationship with mortality risk. Deaths occurred among 10 (25.0%), 14 (35.0%) and 26 (65.0%) participants across T1, T2 and T3, respectively (*P*‐trend = 0.001). In Model 1, participants in the highest SFA tertile (>22.2 g/day) exhibited significantly elevated mortality (HR = 3.56; 95% CI: 1.63–7.75; *P*‐trend = 0.001). This association intensified in Model 2 (HR = 10.4; 95% CI: 2.91–22.43; *P*‐trend = 0.005) and remained robust in the fully adjusted Model 3 (HR = 8.2; 95% CI: 1.33–20.53; *P*‐trend = 0.014). The middle tertile showed non‐significant trends across all models.

#### 3.3.2 MUFAs

MUFA intake was not significantly associated with mortality risk across any analytical model. The number of deaths was 20 (50.0%), 15 (37.5%) and 15 (37.5%) in T1, T2 and T3, respectively (*P*‐trend = 0.559). HRs remained non‐significant in Model 1 (HRT3 vs. T1 = 0.82; 95% CI: 0.39–1.69; *P*‐trend = 0.631), Model 2 (HRT3 vs. T1 = 0.64; 95% CI: 0.21–1.89; *P*‐trend = 0.418) and Model 3 (HRT3 vs. T1 = 0.57; 95% CI: 0.13–2.45; *P*‐trend = 0.470).

#### 3.3.3 PUFAs

Total PUFA intake was not significantly associated with mortality in any model, with 15 (37.5%), 14 (35.0%) and 21 (52.5%) deaths occurring across T1, T2 and T3, respectively (*P*‐trend = 0.212). All three models yielded non‐significant results (Model 1: HRT3 vs. T1 = 1.02, 95% CI: 0.50–2.06, *P*‐trend = 0.932; Model 2: HRT3 vs. T1 = 1.74, 95% CI: 0.59–5.03, *P*‐trend = 0.414; Model 3: HRT3 vs. T1 = 1.48, 95% CI: 0.37–5.88, *P*‐trend = 0.625).

Similarly, PUFA‐3 (omega‐3 fatty acids) demonstrated no significant associations across models, with 20 (50.0%), 18 (45.0%) and 12 (30.0%) deaths in T1, T2 and T3, respectively (*P*‐trend = 0.152). HRs remained non‐significant in all models (Model 1: HRT3 vs. T1 = 0.8, 95% CI: 0.42–1.52, *P*‐trend = 0.404; Model 2: HRT3 vs. T1 = 0.54, 95% CI: 0.22–1.34, *P*‐trend = 0.227; Model 3: HRT3 vs. T1 = 0.78, 95% CI: 0.24–2.49, *P*‐trend = 0.346).

In contrast, PUFA‐6 (omega‐6 fatty acid) intake exhibited a strong positive association with mortality. Deaths increased markedly across tertiles: 6 (15.0%), 15 (37.5%) and 29 (72.5%) in T1, T2 and T3, respectively (*P*‐trend = 0.001). The highest tertile (>4.8 g/day) demonstrated significantly elevated mortality risk across all models: Model 1 (HR = 3.46; 95% CI: 1.4–8.4; *P*‐trend = 0.001), Model 2 (HR = 4.03; 95% CI: 1.45–11.2; *P*‐trend = 0.005) and Model 3 (HR = 5.24; 95% CI: 1.1–26.44; *P*‐trend = 0.014).

### 3.4 Survival analysis

Kaplan–Meier survival curves stratified by fatty acid intake tertiles are displayed in Figures 1, 2, 3, 4. For total fat intake (Figure 1), the 5‐year cumulative survival rates were 51%, 49% and 40% across T1, T2 and T3, respectively (log‐rank *P* = 0.003), demonstrating significantly reduced survival in the highest intake tertile. Similarly, SFA intake (Figure 2) showed progressively declining survival rates of 52%, 50% and 36% across tertiles (log‐rank *P *< 0.001).

**FIGURE 1:**
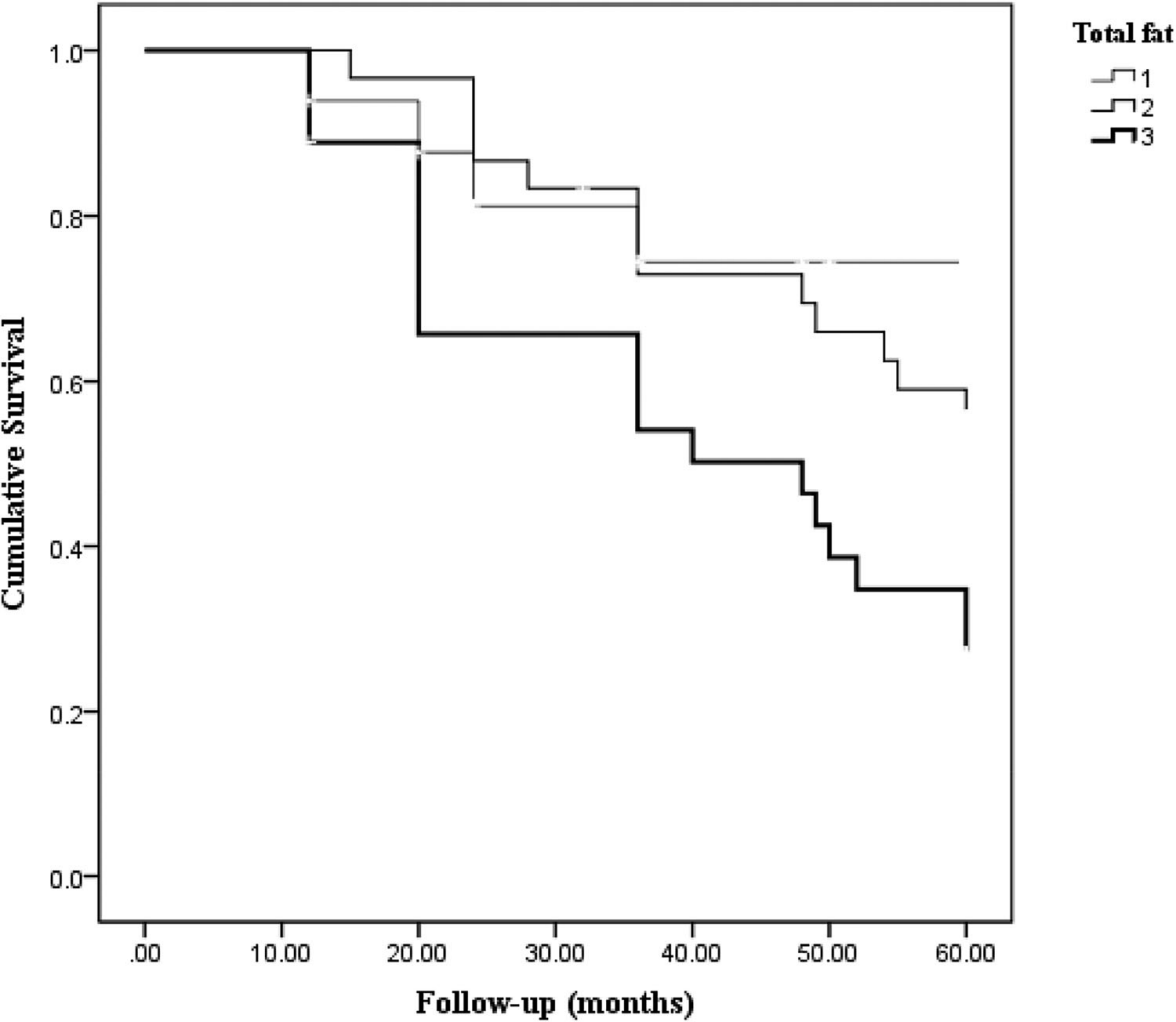
Kaplan–Meier survival curve for death among cirrhotic patients stratified by tertiles of total fat intake (grams per day). The 5‐year survival rate among patients across tertiles was 51%, 49% and 40%, respectively (log‐rank test for homogeneity, *P* = 0.003).

**FIGURE 2:**
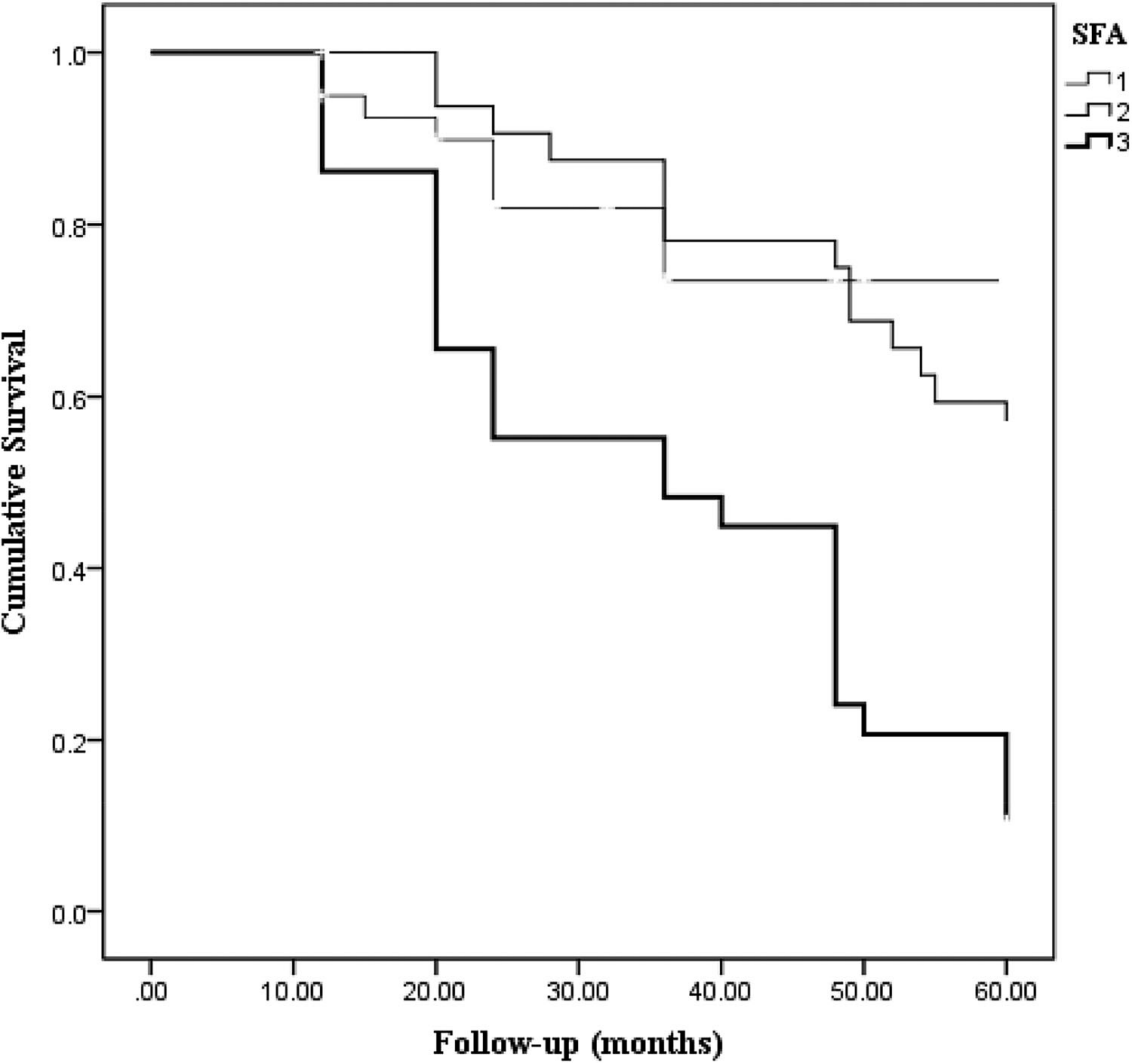
Kaplan–Meier survival curve for death among cirrhotic patients stratified by tertiles of dietary SFA intake (grams per day). The 5‐year survival rate among patients across tertiles was 52%, 50% and 36%, respectively (log‐rank test for homogeneity, *P *< 0.001).

**FIGURE 3:**
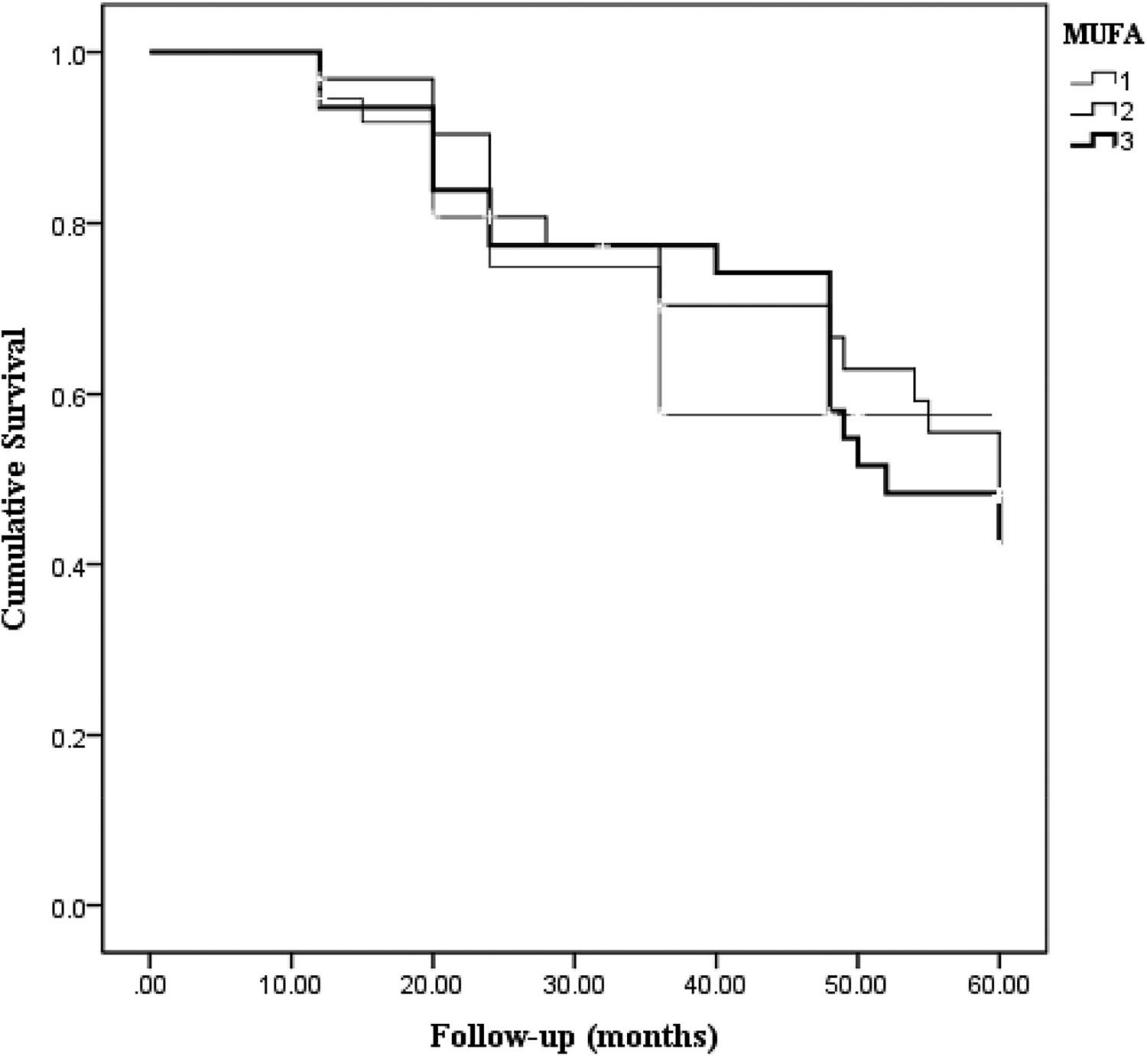
Kaplan–Meier survival curve for death among cirrhotic patients stratified by tertiles of MUFA intake (grams per day). The 5‐year survival rate among patients across tertiles was 45%, 48% and 47%, respectively (log‐rank test for homogeneity, *P* = 0.759).

**FIGURE 4:**
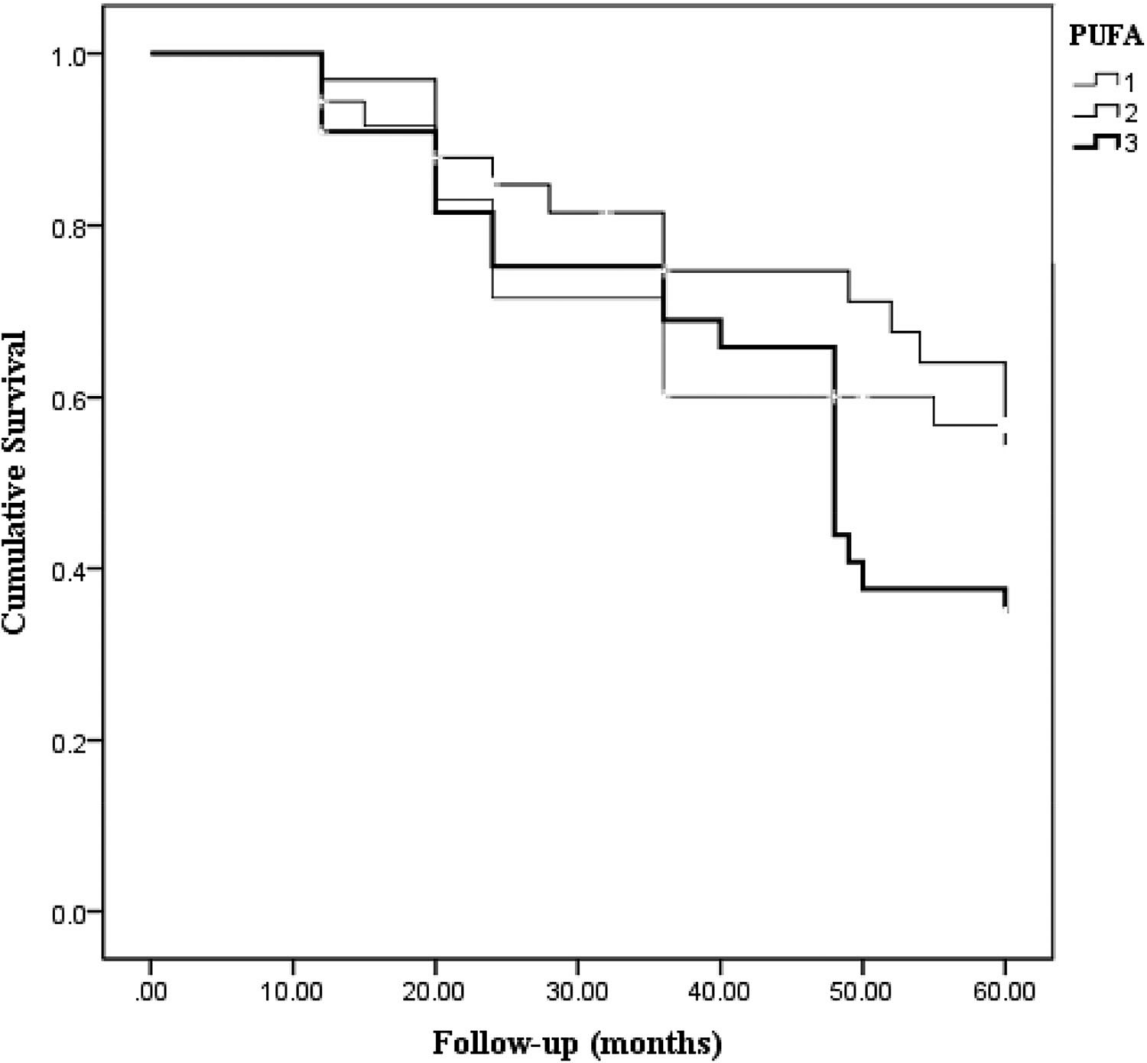
Kaplan–Meier survival curve for death among cirrhotic patients stratified by tertiles of PUFA (grams per day). The 5‐year survival rate among patients across tertiles was 45%, 50% and 44%, respectively (log‐rank test for homogeneity, *P* = 0.207).

Conversely, MUFA intake (Figure 3) exhibited nearly equivalent survival across tertiles (45%, 48% and 47% for T1, T2 and T3, respectively; log‐rank *P* = 0.759), indicating no differential survival based on intake levels. Total PUFA intake (Figure 4) also demonstrated no significant survival differences, with 5‐year rates of 45%, 50% and 44% across tertiles (log‐rank *P* = 0.207).

## 4 DISCUSSION

To our knowledge, this represents the first prospective cohort investigation systematically examining the association between habitual dietary fatty acid intake patterns and all‐cause mortality risk among patients with established liver cirrhosis. Our principal findings demonstrate that higher intakes of total dietary fat, SFAs and omega‐6 PUFAs were independently associated with significantly elevated mortality risk after comprehensive adjustment for demographic, lifestyle and clinical confounders. Conversely, dietary intakes of MUFAs, total PUFAs and omega‐3 PUFAs showed no significant associations with survival outcomes in this cirrhotic population. Adjustment for MELD and Child–Pugh scores strengthened the associations between fat intake and mortality. This likely reflects negative confounding, as patients with more advanced cirrhosis often consume less dietary fat due to anorexia, malabsorption or dietary restrictions. Controlling for disease severity therefore removed a suppressor effect, revealing a stronger underlying association.

The robust dose–response relationship between SFA intake and mortality observed in our study aligns with accumulating evidence linking SFA to adverse liver‐related outcomes. Although the HRs observed for total fat and saturated fat intake were relatively high compared with typical nutritional epidemiology estimates, model diagnostics did not indicate over‑adjustment or instability. Nonetheless, these effect sizes should be interpreted with caution, and replication in larger cohorts is warranted. The early separation of survival curves for SFA likely reflects long‑standing dietary exposure and metabolic vulnerability that pre‑dated cohort enrolment, rather than an acute post‑baseline effect. MELD and Child–Pugh scores capture hepatic synthetic function but do not reflect metabolic, inflammatory or nutritional pathways through which saturated fat may influence mortality risk. Recent large‐scale investigations have identified similar associations; a UK Biobank study of over 250,000 participants demonstrated that elevated plasma SFA levels were associated with substantially increased risks of both hepatocellular carcinoma and chronic liver disease mortality (Liu et al., 2024). These findings are consistent with a proposed mechanistic framework in which SFAs may exacerbate hepatic pathology through several interconnected pathways. Experimental models suggest that SFAs can promote *de novo* lipogenesis, induce endoplasmic reticulum stress, contribute to mitochondrial dysfunction and activate inflammatory cascades (Arrese et al., 2016; Song et al., 2023), although the extent to which these pathways operate in human cirrhosis, as opposed to preclinical models, remains to be established. The paradoxical observation that saturated fat may be protective in alcohol‐related liver disease (Kirpich et al., 2016; Nanji & French, 1986), while appearing deleterious in our mixed‐aetiology cohort, suggests that fatty acid–liver interactions may vary substantially by underlying disease mechanism and require further investigation.

The marked positive association between omega‐6 PUFA intake and mortality represents a particularly novel and clinically significant finding. While omega‐6 fatty acids, particularly linoleic acid, have demonstrated protective associations in some general population cohorts (Zhuang et al., 2019), our results suggest a fundamentally different relationship in established cirrhosis. This apparent discordance may be mechanistically explicable through several pathways. Omega‐6 fatty acids serve as precursors for arachidonic acid‐derived eicosanoids, including prostaglandins and leukotrienes, which are generally considered to have pro‐inflammatory and pro‐coagulatory properties (Dennis & Norris, 2015). It is plausible, though not directly demonstrated in cirrhotic patients, that excessive omega‐6 intake could amplify these pathological processes in this population. Some investigations have reported correlations between elevated omega‐6/omega‐3 ratios and markers of oxidative stress and hepatic fibrosis progression (Araya et al., 2004), though causal direction cannot be established from such data. Furthermore, a prospective analysis of plasma fatty acids and cirrhosis complications revealed that while omega‐6 PUFAs showed inverse associations overall, this protective effect diminished and reversed beyond certain threshold levels, suggesting a U‐shaped relationship (Guo et al., 2024). Our dietary data may reflect intake levels exceeding optimal thresholds, thereby triggering pro‐inflammatory cascades rather than conferring metabolic benefits.

The absence of significant associations for MUFA, total PUFA and omega‐3 PUFA warrants careful interpretation. Experimental models have suggested hepatoprotective properties for MUFAs, particularly in promoting hepatic histological recovery and reducing steatosis (Fernandez et al., 1997; Moreira et al., 1995). However, observational human studies have yielded inconsistent results, with some investigations reporting elevated plasma MUFA levels associated with increased chronic liver disease mortality (Liu et al., 2024). The heterogeneity in MUFA sources may partially explain these divergent findings, as animal MUFAs have been associated with higher mortality in large prospective cohorts (Zhuang et al., 2019). Regarding omega‐3 fatty acids, while cirrhotic livers demonstrate marked depletion of docosahexaenoic acid and omega‐3 supplementation has shown benefits in non‐alcoholic fatty liver disease (NAFLD) populations (Lee et al., 2020; Lu et al., 2016), our null findings may reflect several considerations. First, baseline omega‐3 intake in our cohort was relatively low, potentially limiting our ability to detect protective associations at higher intake levels. Second, the relationship between omega‐3 fatty acids and cirrhosis outcomes may be non‐linear, with protective effects manifesting only above specific threshold intakes (Guo et al., 2024). Third, as one study demonstrated, plasma omega‐3 and omega‐6 levels showed no association with cirrhosis severity or systemic inflammation (Schwarzkopf et al., 2019), suggesting that individual fatty acid classes may exert less influence than their relative balance or interaction with the specific inflammatory milieu of advanced liver disease.

The strengthening of the HRs with progressive adjustment underscores the importance of considering total caloric context when evaluating fatty acid–health relationships. This observation suggests that the deleterious effects of certain fatty acids may be partially mediated through or modified by overall energy balance and metabolic status, consistent with contemporary understanding of nutrient‐disease interactions.

Several biological mechanisms have been proposed to link dietary fatty acid composition to cirrhosis mortality, though most evidence derives from preclinical or non‐cirrhotic models. In such models, SFAs have been shown to induce hepatocyte lipotoxicity through ceramide accumulation, with downstream effects on apoptotic signalling, insulin resistance and mitochondrial function (Musso et al., 2018; Yki‐Järvinen et al., 2021); whether these pathways are similarly operative in decompensated cirrhosis is not established. These lipotoxic effects are amplified in the setting of pre‐existing hepatic injury, where regenerative capacity is impaired and inflammatory responses are dysregulated. In addition, saturated fats have been shown in experimental settings to stimulate transforming growth factor‐β production, a pathway implicated in hepatic fibrogenesis (Carmiel‐Haggai et al., 2003), though direct evidence in cirrhotic patients is lacking. It has been hypothesized that pro‐inflammatory omega‐6‐derived eicosanoids could contribute to portal hypertension, bacterial translocation and decompensation events via effects on vascular permeability and coagulation (Araya et al., 2004; Dennis & Norris, 2015); however, this remains a hypothesis that has not been directly tested in cirrhotic cohorts. Additionally, altered fatty acid composition affects membrane fluidity, receptor function and intracellular signalling in hepatocytes, Kupffer cells and hepatic stellate cells (Juárez‐Hernández et al., 2015).

Insulin resistance may represent another mechanistic link between dietary fat and clinical outcomes, although this connection remains speculative in this population. Evidence from other settings suggests SFAs may exacerbate insulin resistance more than unsaturated fats (Vessby et al., 2001); insulin resistance has in turn been associated with activation of the insulin‐like growth factor‐1 pathway, a pathway implicated in hepatocyte proliferation and hepatocarcinogenesis in some studies (Szablewski, 2024), though a direct chain of causation from dietary fat through this pathway to mortality in cirrhosis has not been demonstrated. Furthermore, insulin resistance drives elevated pro‐inflammatory cytokines including tumour necrosis factor‐alpha (TNF‐α) and interleukin (IL)‐6, which perpetuate hepatic inflammation and fibrosis progression (Park et al., 2010). A further, more speculative dimension involves the interplay between dietary fatty acids, gut microbiota composition and intestinal barrier integrity. Specific fatty acid profiles have been shown to modulate bacterial populations and endotoxin translocation in some studies (Kirpich et al., 2016; Schulthess et al., 2019), and these processes are recognized contributors to hepatic inflammation in cirrhosis; however, whether dietary fatty acid intake influences cirrhosis mortality through this specific pathway has not been directly studied.

Our findings both complement and extend the limited existing literature on dietary fats and liver disease outcomes. A recent Swedish cohort study examining dietary substitutions found no clear associations between replacing saturated fat with other macronutrients and risk of NAFLD cirrhosis or hepatocellular carcinoma (Fridén et al., 2024), potentially reflecting differences in study populations (general population prevention vs. established cirrhosis prognosis), disease mechanisms and dietary assessment methodologies. Conversely, studies examining circulating (rather than dietary) fatty acids have more consistently demonstrated adverse associations for saturated fats and protective associations for polyunsaturated fats with liver disease outcomes (Guo et al., 2024; Liu et al., 2024), though the correlation between dietary intake and plasma concentrations is imperfect and modified by endogenous metabolism. The discordance between our PUFA‐6 findings and those from biomarker studies may reflect the distinction between long‐term habitual dietary exposure (captured by FFQ) versus more proximal metabolic states (reflected in plasma measurements), as well as the complex interplay between dietary intake, hepatic synthesis and oxidative degradation of fatty acids in cirrhotic patients.

Within nutritional hepatology, current guidelines for cirrhosis recommend total fat intake of 10–20% or up to 30% of total calories, with saturated fat limited to below 10% (Javaid et al., 2021; Plauth et al., 2019). Our findings provide empirical support for these recommendations while highlighting the potential importance of omega‐6/omega‐3 balance, which is not explicitly addressed in most existing guidelines. The observation that omega‐6 fatty acids, often considered heart‐healthy in general population contexts, may be problematic in cirrhotic patients underscores the need for disease‐specific dietary recommendations rather than universal prescriptions.

### 4.1 Implications for public health and clinical practice

The findings from this investigation carry substantial implications for nutritional management of cirrhotic patients and warrant integration into clinical practice guidelines. First and foremost, our results strongly support limiting SFA intake in patients with liver cirrhosis. Given the robust and consistent association observed across all analytical models (HRs ranging from 3.56 to 10.4), dietary counselling may consider emphasizing reduced consumption of SFA‐rich foods, including animal fats, full‐fat dairy products, processed meats and certain plant oils high in saturated fats. This recommendation aligns with Mediterranean dietary patterns, which derive most energy from unsaturated fats and limit saturated fat to below 10% of total calories.

Second, the striking association between omega‐6 PUFA intake and mortality necessitates careful consideration of the omega‐6/omega‐3 fatty acid balance rather than focusing solely on total fat or fat classes in isolation. Current Western diets typically exhibit omega‐6/omega‐3 ratios ranging from 10:1 to 20:1, substantially higher than evolutionary ratios of approximately 1:1 to 4:1 (Simopoulos, 2008). Our findings suggest that cirrhotic patients may represent a population in whom optimization of this ratio warrants further investigation: (1) moderating omega‐6 intake by limiting consumption of corn oil, sunflower oil, safflower oil and products containing these oils; and (2) potentially increasing omega‐3 sources such as fatty fish, walnuts, flaxseed and marine‐derived supplements, though our study cannot definitively establish benefit for the latter given the null findings for omega‐3 intake. If confirmed in future interventional studies, these findings may have implications for dietary counselling approaches that help patients identify and modify specific food sources rather than abstract fatty acid categories.

Third, these results underscore the critical importance of personalized nutritional assessment and intervention in cirrhosis care. Registered dietitians specializing in hepatology should conduct comprehensive dietary evaluations for all cirrhotic patients, with particular attention to fat quality and quantity. Given that malnutrition affects 50–90% of cirrhotic patients depending on severity (Tandon et al., 2017), and that sarcopenia independently predicts mortality (Bhanji et al., 2017), nutritional optimization represents a modifiable prognostic factor that deserves prominence in multidisciplinary cirrhosis management. The potential role of fatty acid profiling in nutritional assessment warrants further investigation.

Fourth, from a public health perspective, these findings highlight the need for disease‐specific dietary guidelines that may diverge from general population recommendations. While omega‐6 PUFAs have been associated with cardiovascular benefits and are encouraged in healthy populations (Farvid et al., 2014), our results suggest they may require restriction in cirrhotic patients. Similarly, the complex relationship between saturated fat and alcoholic versus metabolic liver disease (Kirpich et al., 2016; Nanji & French, 1986) suggests that uniform dietary prescriptions may be inadequate. Healthcare systems should invest in specialized nutritional support services for chronic liver disease clinics, and medical education should emphasize the nuanced, disease‐specific nature of dietary fat recommendations.

Fifth, these findings may inform future nutritional intervention trials. The magnitude of associations observed suggests that dietary modification could meaningfully impact mortality risk. Randomized controlled trials (RCTs) testing dietary interventions that simultaneously limit saturated and omega‐6 fatty acids while potentially increasing omega‐3 intake (analogous to Mediterranean or anti‐inflammatory dietary patterns) would provide definitive evidence for causality and inform evidence‐based guidelines. Such trials should incorporate not only mortality endpoints but also intermediate outcomes including decompensation events, hepatic encephalopathy episodes, infection rates and quality of life measures.

Finally, given the global increase in cirrhosis prevalence (Huang et al., 2023) and the relatively simple, low‐cost nature of dietary interventions compared with pharmacological therapies, population‐level strategies to promote optimal fat intake in at‐risk populations warrant consideration. Public health campaigns targeting individuals with chronic liver disease, patient education materials emphasizing practical dietary modifications, and healthcare provider training programmes on therapeutic nutrition in hepatology could substantially improve population outcomes if our findings are confirmed in additional cohorts and intervention studies.

### 4.2 Strengths and limitations, and future research directions

This investigation possesses several methodological strengths that enhance confidence in the findings. First, to our knowledge, this represents the inaugural prospective cohort study explicitly designed to examine dietary fatty acid intake in relation to mortality among patients with established liver cirrhosis, addressing a critical gap in the nutritional hepatology literature. The prospective design with systematic 60‐month follow‐up minimizes recall bias and establishes appropriate temporality for causal inference. Second, we employed comprehensive multivariable adjustment strategies, sequentially controlling for demographic factors, lifestyle behaviours, anthropometric measures and disease severity indicators including MELD score and Child–Pugh classification. This rigorous approach to confounding control, particularly the inclusion of validated prognostic scores, substantially reduces the likelihood that observed associations reflect residual confounding by disease severity. Third, the detailed dietary assessment utilizing a validated, population‐specific 168‐item FFQ administered before substantial disease‐related dietary modifications permits characterization of habitual intake patterns rather than intake altered by symptoms or medical advice. Fourth, our investigation examined not only total fat but also constituent fatty acid subclasses including SFA, MUFA, total PUFA, omega‐3 and omega‐6 fatty acids, enabling nuanced evaluation of specific fat types rather than treating dietary fat as a monolithic entity. This granular approach permits clinically actionable conclusions about which specific fatty acids warrant dietary modification. Fifth, the use of energy‐adjusted fatty acid intakes using the residual method accounts for total caloric consumption, isolating the specific effects of fat quality from quantity. Sixth, the application of multiple statistical approaches including Cox proportional hazards models with trend tests and Kaplan–Meier survival analyses provides complementary perspectives on the data and strengthens inference.

Nevertheless, several limitations warrant acknowledgment and consideration when interpreting our findings. First, this was an exploratory (hypothesis‐generating) study and no a priori sample size calculation was performed because of the limited availability of eligible patients within this prospective cohort. Consequently, the relatively modest sample size may have limited statistical power, particularly for subgroup analyses and for detecting modest associations. The wide CIs observed for several estimates reflect this limitation, and our ability to detect meaningful differences for MUFA and omega‑3 fatty acids may have been constrained. Larger multicentre cohorts are needed to provide more precise effect estimates and enable adequately powered subgroup analyses by cirrhosis aetiology, disease severity, sex and genetic factors. Also, the number of events per stratum was limited, which may affect the stability and precision of HR estimates. Although we used a sequential modelling strategy and restricted covariates to those with strong biological justification, the possibility of model overfitting cannot be excluded. Proportional hazards testing using Schoenfeld residuals did not indicate major violations; however, we acknowledge that these tests have reduced reliability in small samples. Therefore, the findings should be interpreted with caution and considered hypothesis‑generating until replicated in larger cohorts. Second, dietary assessment relied on a single FFQ administration at baseline, which does not capture dietary changes during follow‐up that may occur due to disease progression, medical counselling or other factors. Repeated dietary assessments would enable examination of temporal relationships and assessment of whether dietary modifications during follow‐up influence outcomes. Third, although we employed a validated FFQ, all self‐reported dietary assessments are susceptible to measurement error and recall bias. Although energy intake was included in all multivariable models, we did not adjust for total protein intake. Given the importance of protein‑energy malnutrition and sarcopenia in cirrhosis, residual confounding by protein intake cannot be excluded. The magnitude of such errors likely does not differ systematically across outcome groups, which would tend to bias associations toward the null, suggesting our findings may underestimate true associations. Nevertheless, objective biomarkers of fatty acid status, such as plasma phospholipid or red blood cell membrane fatty acid composition, would provide complementary, less error‐prone exposure assessment. Fourth, the observational study design precludes definitive causal inference despite comprehensive confounding control. Unmeasured confounders, including unmeasured dietary components, physical activity patterns, medication use, socioeconomic factors, or genetic variants affecting fatty acid metabolism may partially explain observed associations. Fifth, our cohort was recruited from two tertiary‐care centres in Tehran, which may limit generalizability to other geographical regions, ethnicities or healthcare settings. Dietary patterns, fat sources and disease characteristics may differ substantially across populations, and external validation in diverse cohorts is essential. Sixth, we did not assess specific food sources of fatty acids, which would provide more actionable dietary guidance. Future studies should examine whether associations differ for plant versus animal fat sources, specific oils, or dietary patterns. Seventh, our investigation could not assess dose–response relationships beyond tertile comparisons, and optimal intake thresholds for various fatty acids remain undefined. Eighth, we did not have access to biological markers such as plasma fatty acid profiles, inflammatory cytokines, oxidative stress indicators, or lipid panels. Biomarkers could have helped clarify mechanistic pathways linking saturated fat and omega‑6 intake to mortality, and could have reduced potential measurement error inherent in self‑reported dietary data. Therefore, our findings should be interpreted as associations based on reported habitual intake rather than biologically verified fatty acid status.

These limitations illuminate critical directions for future research that could substantially advance the field. First, large‐scale, multicentre prospective cohorts with comprehensive dietary assessment, biomarker measurement and genetic data are needed to provide robust, generalizable estimates of fatty acid‐mortality relationships across diverse cirrhotic populations. Such cohorts should specifically enrol sufficient participants across different aetiologies to permit well‐powered subgroup analyses, as mechanisms and optimal dietary interventions may differ by underlying cause. Second, RCTs testing dietary interventions targeting fatty acid composition are urgently needed to establish causality and guide evidence‐based recommendations. Such trials might compare Mediterranean dietary patterns (emphasizing unsaturated fats from olive oil, nuts and fish while limiting saturated fats) against standard dietary advice in cirrhotic patients, with endpoints including mortality, decompensation events and quality of life. Given ethical considerations of potentially harmful interventions, trials would likely focus on restricting harmful fats (SFA, excess omega‐6) rather than increasing them. Third, mechanistic investigations employing metabolomics, lipidomics and inflammatory marker profiling could elucidate the pathways through which specific fatty acids influence outcomes in cirrhosis, potentially identifying intermediate targets for intervention. Integration of multi‐omic approaches (transcriptomics, proteomics, metabolomics) with detailed dietary data would provide unprecedented insight into nutrient–disease interactions. Fourth, research examining gene–diet interactions, particularly involving genes regulating fatty acid metabolism (*FADS1/2*, *ELOVL2*), inflammation (*IL‐6*, *TNF‐α*) and fibrosis (*PNPLA3*, *TM6SF2*) could enable personalized nutritional recommendations based on genetic risk profiles. Fifth, investigations examining the gut microbiome as a mediator of dietary fat effects on liver outcomes represent a promising frontier, as specific fatty acids potently modulate microbial composition, which in turn influences hepatic inflammation, barrier function and metabolism. Sixth, pragmatic implementation science research testing strategies to integrate evidence‐based nutritional counselling into routine cirrhosis care, including electronic screening tools, telehealth dietitian consultations, and patient‐facing educational apps would accelerate translation of research findings into improved population health. Seventh, health economic analyses quantifying the cost‐effectiveness of intensive nutritional interventions in cirrhosis would inform resource allocation decisions and strengthen the case for reimbursement of specialized nutrition services. Finally, longitudinal studies examining bidirectional relationships between disease progression and dietary intake patterns would clarify whether certain dietary changes represent causes or consequences of clinical deterioration, informing interpretation of observational associations.

### 4.3 Conclusion

In conclusion, this exploratory prospective cohort study provides preliminary evidence that higher dietary intakes of SFAs and omega‐6 PUFAs may be associated with increased all‐cause mortality among patients with liver cirrhosis. Given the exploratory nature of the study, the relatively small sample size, and the absence of an a priori sample size calculation, these findings should be considered hypothesis‐generating and interpreted with caution. Confirmation in adequately powered multicentre prospective cohorts and randomized clinical trials is required before definitive clinical recommendations can be made.

## AUTHOR CONTRIBUTIONS

Conceptualization: Zahra Yari and Azita Hekmatdoost. Formal analysis: Zahra Yari. Methodology: Mehdi Saberifiroozi, Fereshteh Pashayee‐Khamene, Behzad Hatami, Saleheh Ahmadzadeh and Sara Karimi. Project administration: Ali Jafari, Ghazaleh Mahdavi‐Mazdeh, Behnaz Narimani and Helia Mardani. Writing—original draft: Ali Jafari, Ghazaleh Mahdavi‐Mazdeh, Behnaz Narimani and Helia Mardani. Writing—review & editing: Zahra Yari and Azita Hekmatdoost. All authors have read and approved the final version of this manuscript and agree to be accountable for all aspects of the work in ensuring that questions related to the accuracy or integrity of any part of the work are appropriately investigated and resolved. All persons designated as authors qualify for authorship, and all those who qualify for authorship are listed.

## CONFLICT OF INTEREST

There are no conflicts of interest related to the study design or its results.

## FUNDING INFORMATION

No funding was received for this work.

## GENERATIVE AI STATEMENT

The authors confirm that no artificial intelligence tools, including large language models (LLMs), were used in the drafting or revision of this manuscript. All content was conceived, written, and approved solely by the authors.

## Data Availability

The datasets analysed in the current study are available from the corresponding author on reasonable request.

## Appendix

**TABLE A1:** Protein intake of participants according to the fat intake tertile

	Tertile of total fat intake			
	T1	T2	T3	P value
Protein (%TEE)	13.85 ± 2.26	14.24 ± 2.36	14.38 ± 2.12	0.653
Protein (g/day)	64.6 ± 16.4	75.2 ± 15.1	77.8 ± 13.1	0.070

Values are means ± SDs

ANOVA test

**TABLE A2:** Sensitivity model adjusted for total protein intake

*P* trend	Tertiles of fat intake			
Total fat				
	T3 (68.9 ≤)	T2 (50.7‐68.9)	T1 (< 50.7)	
0.031	23	16	11	No. of deaths
0.020	3.39 (1.1‐10.7)	0.54 (0.1‐3.37)	ref	Model 3

Cox proportional hazards regression models for estimating HRs and 95% CIs. Adjusted for age and sex, energy intake, BMI, smoking and alcohol, etiology, MELD and child and protein intake
